# Lower-Order Compensation Chain Threshold-Reduction Technique for Multi-Stage Voltage Multipliers

**DOI:** 10.3390/s18041245

**Published:** 2018-04-17

**Authors:** Francesco Dell’ Anna, Tao Dong, Ping Li, Yumei Wen, Mehdi Azadmehr, Mario Casu, Yngvar Berg

**Affiliations:** 1 Institute of Applied Micro-Nano Science and Technology—IAMNST, Chongqing Key Laboratory of Colleges and Universities on Micro-Nano Systems Technology and Smart Transducing, Chongqing Engineering Laboratoryfor Detection, Control and Integrated System, National Research Base of Intelligent Manufacturing Service, Chongqing Technology and Business University, Nan’an District, Chongqing 400067, China; francescodellanna@email.ctbu.edu.cn; 2 Faculty of Engineering, Science and Maritime Studies, Department of Microsystems, Campus Vestfold, Høgskolen i Sørøst-Norge, 235 3603 Kongsberg, Norway; mehdi.azadmehr@usn.no (M.A.); yngvar.berg@usn.no (Y.B.); 3 Department of Instrumentation, School of Electronic Information and Electric Engineering, Shanghai Jiao Tong University, Shanghai 200240, China; liping_sh@sjtu.edu.cn (P.L.); yumei.wen@sjtu.edu.cn (Y.W.); 4 Department of Electronics and Telecommunications (DET), Politecnico di Torino, Corso Duca degli Abruzzi No. 24, 10129 Torino, Italy; mario.casu@polito.it

**Keywords:** voltage multiplier, threshold compensation technique, wireless sensing node (WSN), energy harvesting

## Abstract

This paper presents a novel threshold-compensation technique for multi-stage voltage multipliers employed in low power applications such as passive and autonomous wireless sensing nodes (WSNs) powered by energy harvesters. The proposed threshold-reduction technique enables a topological design methodology which, through an optimum control of the trade-off among transistor conductivity and leakage losses, is aimed at maximizing the voltage conversion efficiency (VCE) for a given ac input signal and physical chip area occupation. The conducted simulations positively assert the validity of the proposed design methodology, emphasizing the exploitable design space yielded by the transistor connection scheme in the voltage multiplier chain. An experimental validation and comparison of threshold-compensation techniques was performed, adopting 2N5247 N-channel junction field effect transistors (JFETs) for the realization of the voltage multiplier prototypes. The attained measurements clearly support the effectiveness of the proposed threshold-reduction approach, which can significantly reduce the chip area occupation for a given target output performance and ac input signal.

## 1. Introduction

Wireless sensor nodes (WSNs) are valuable burgeoning technologies which have been ubiquitously adopted in a wide range of applications, such as building monitoring, environment control, and military systems [1]. The upsurging need for passive and autonomous WSNs demands advanced technology to scavenge energy from the surrounding environment [2,3,4,5]. Energy harvesting is a promising solution, and can be employed to satisfy the power demand of WSNs.

**Figure 1:**
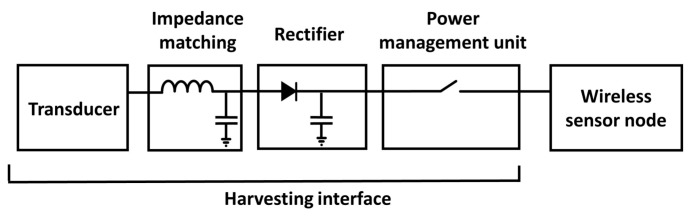
General architecture of a wireless sensor node (WSN) powered by an energy harvester interface.

Conventional energy harvesters adopt, in sequence: (a) a transducer to scavenge energy from the environment, (b) an input matching network to minimize the power reflection, (c) a rectifier to condition the input signal into a constant supply voltage, and (d) a power management unit (PMU) to store and regulate the harvested energy, as shown in Figure 1.

Due to the low power density of harvesting transducers (<10 mW/cm${}^{2}$ [6]), the input voltage levels attainable at the matching block output are often below the operational threshold voltage of the WSN. Therefore, customary power conditioning interfaces for energy harvesters are suitably designed to up-convert and regulate the input voltage coherently with the specifications of the target WSN.

Voltage multipliers are rectifiers employed to generate a constant output voltage that exceeds the maximum peak-to-peak voltage of the input ac signal. Unlike conventional dc–dc up-converters relying on inductive components to boost the output voltage, voltage multipliers comprise only diodes or diode-connected transistors and capacitors, thereby enabling a better silicon integration [7,8]. Saif et al. [9] proposed a quantitative comparison between capacitor- and inductor-based switched converters. In [9], two dc–dc converters were designed and prototyped adopting the 1 μm Silicon on Insulator (SOI) fabrication process. Both converters were designed to step up the input voltage from 5 V up to 400 V. One converter was implemented with a multistage boost architecture, whereas the other converter was implemented adopting charge pumps. The boost converter outperformed the charge-pump-based architecture in terms of power conversion efficiency, output current, and silicon area; however, in applications where limited performances can be tolerated and packaged volume is a stringent requirement, voltage multipliers substantiate a better implementation.

**Figure 2:**
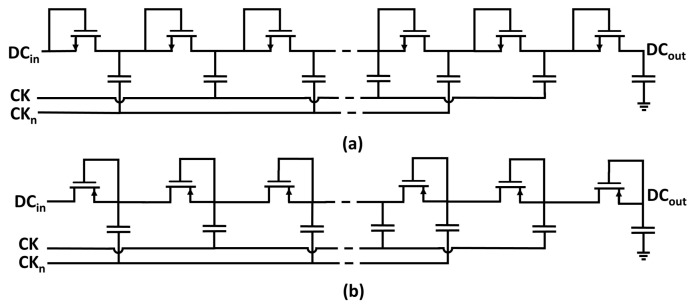
Circuit schematic of (**a**) Negative-Channel Metal Oxide Semiconductor (NMOS) and (**b**) Positive- Channel Metal Oxide Semiconductor (PMOS) implementations of a multi-stage Dickson rectifier.

Traditional on-chip multipliers are based on the circuit topology proposed by Dickson in [10], where two non-interleaving clock signals, used as input ac source, are coupled with parallel capacitors interconnected by diode-connected transistors, as depicted in Figure 2. Dickson-based voltage multipliers are conventionally adopted to program electrically erasable programmable read-only memories (EEPROMs) [11,12,13] or to reduce the leakage power and the fetch access time in static random-access memories (SRAMs) and in dynamic random-access memories (DRAMs) [14].

The continuous progress observed in complementary metal–oxide–semiconductor (CMOS) technology scaling extended the range of possible applications in which voltage multipliers can be adopted [15]. As result, voltage multipliers can be operated at higher input frequencies and at lower peak-to-peak ac input voltages. Under the aforementioned operating conditions, rather complex traditional multiplier topologies, which profitably exploit on-chip clocks to generate a constant output voltage, are outperformed by simpler rectification designs, when considering the attainable power conversion efficiency (PCE, defined as the ratio between the input output power during steady state operation) and voltage conversion efficiency (VCE), estimated considering the ratio between the output dc voltage and the peak-to-peak amplitude of the ac input voltage.

**Figure 3:**
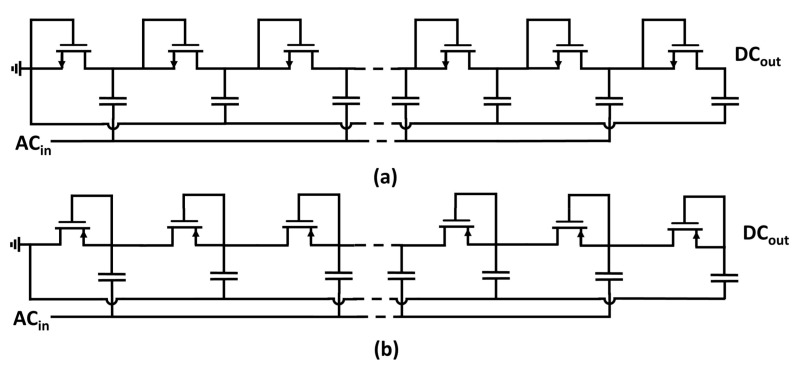
Circuit diagram of (**a**) NMOS and (**b**) PMOS implementations of a multi-stage Dickson voltage multiplier in the case of a single power source.

The Dickson topology can also be readopted to rectify a single ac signal (e.g., RF applications) by grounding the dc input and one of the two clock inputs [16,17], as shown in Figure 3.

The limitation imposed by the Dickson multiplier becomes increasingly noticeable as the input ac voltage approaches the transistor threshold voltage employed in the multiplication chain, which is operated in cutoff mode for most of the conduction period. This effect is referred to in the literature as the input dead-zone of voltage multipliers [18].

**Figure 4:**
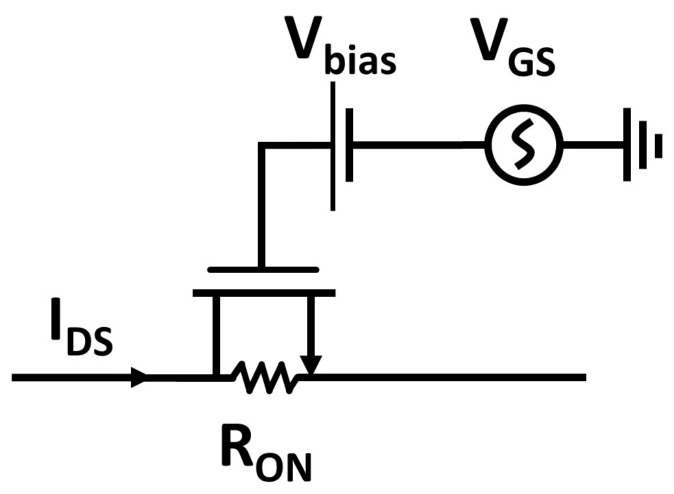
Circuit schematic of an NMOS transistor with a superposed dc bias voltage.

Threshold-compensation techniques [19] inevitably entail a physical chip area overhead of the integrated circuit (IC), and allow a reduction of the input dead-zone imposed by the physical characteristics of non-ideal MOS transistors (e.g., channel length, insulator thickness). The threshold reduction (in this article, the expression threshold reduction refers only to boosting techniques which are used to either increase or decrease the gate voltage of MOS transistors) is achieved by supplying a bias offset voltage at the transistor gates, resulting in an enhanced transistor conductivity (i.e., a reduced on-resistance ($R_{ON}$)), which, considering Negative-Channel Metal Oxide Semiconductor (NMOS) transistors operated in saturation, as shown in Figure 4, can be expressed as [20]:

(1) $$R_{ON}=\frac{2L_{n}V_{DS}}{\mu_{n}C_{OX}W_{n}{\left(V_{GS}-V_{TH}+V_{bias}\right)}^{2}\left(1-\lambda V_{DS}\right)},$$

where $V_{TH}$ is the threshold voltage of a transistor, $V_{GS}$ is the gate-to-source voltage, $C_{OX}$ is the oxide capacitance, $L_{n}$ and $W_{n}$ are, respectively, the channel length and width, λ is the channel-length modulation parameter, and $\mu_{n}$ is the electron mobility.

The direct consequence of a threshold voltage reduction is the increased leakage current, which might eventually yield a degradation of the overall PCE.

Therefore, this paper introduces a topological design methodology aimed at maximizing the VCE through an optimum trade-off among transistor conductivity and leakage losses for a given input signal and chip area occupation.

This paper is organized as follows. Section 2 explains the trade-off between the bias voltage and the total number of stages for a multi-stage voltage multiplier. Section 3 provides an overview on prior-art $V_{TH}$-compensation approaches. Section 4 presents the proposed threshold-cancellation technique. Section 5 compares the state-of-the-art $V_{TH}$-reduction techniques with the proposed design methodology. Section 6 shows and discusses the attained measurements. Section 7 concludes the paper.

## 2. Overview of Passive Threshold-Compensation Techniques

The multiplication efficiency attained by the Dickson rectifier exploiting on-chip clock signals as input power source does not depend on the number of multiplier stages [10]. Hence, IC implementations of voltage multipliers are customary based on the Dickson topology [19]. The pumping node voltage expression of the Dickson multiplier is given by [21]:

(2) $$V_{n}=n\left[\frac{C}{C+C_{P}}V_{AC}-\frac{I_{OUT}}{f(C+C_{P})}-V_{TH}\right]-V_{TH},$$

where *n* is the number of the considered multiplication stage, $V_{AC}$ is the input ac peak-to-peak amplitude voltage, *f* is the input frequency, $I_{OUT}$ is the average current supplied to the load, *C* is the capacitance value for the coupling capacitors, and $C_{P}$ is the strain capacitance relative to each multiplication stage. Equation (2) was derived neglecting the nonlinear voltage–current characteristic pertaining to the metal–oxide–semiconductor field-effect transistor (MOSFET) model. When considering the circuit topology shown in Figure 3, the input ac signal is injected only in half of the available pumping cells; hence, the rectifier attains a multiplication stage after two ensuing pumping nodes. Therefore, the output voltage behavior can be expressed as:

(3) $$V_{OUT}=\frac{N}{2}\left[\frac{C}{C+C_{P}}V_{AC}-\frac{I_{OUT}}{f(C+C_{P})}-V_{TH}\right]-V_{TH},$$

where *N* is the total number of pumping nodes. Equation (3) entails a minimum input voltage condition given by:

(4) $$V_{AC}>\frac{C+C_{P}}{C}\frac{N+2}{N}V_{TH}+\frac{I_{OUT}}{fC}$$

to be fulfilled in order to attain a positive output voltage. Whenever the peak-to-peak amplitude of the input signal does not fulfill the condition expressed in Equation (4), the problem of the input dead-zone of voltage multipliers is incurred [18].

At low power levels, the Dickson multiplier topology attains performance rigidly related to the threshold voltage of transistors in the rectification chain.

The problem of the input dead-zone can be reduced by making specific technological choices like Schottky diodes, floating-gate transistors, or zero-$V_{TH}$ transistors as pumping devices, as proposed in [22,23,24,25]. The choice of a non-standard fabrication process intrinsically entails an enhanced fabrication cost for the rectifier.

**Figure 5:**
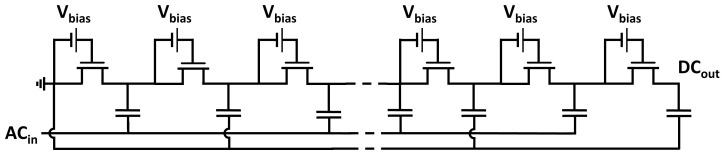
Circuit schematic of a threshold-compensation technique with ideal dc bias voltages.

Threshold-compensation techniques, implemented using standard CMOS technology, can be adopted to provide a bias voltage at the gate terminal of the transistors, as depicted in Figure 5. One implementation of this technique can be found in [26], where an external battery was employed to provide the required bias voltage. The proposed system is not passive; therefore, the threshold-compensation technique in [26] inherently imposes constraints on the possible target application.

**Figure 6:**
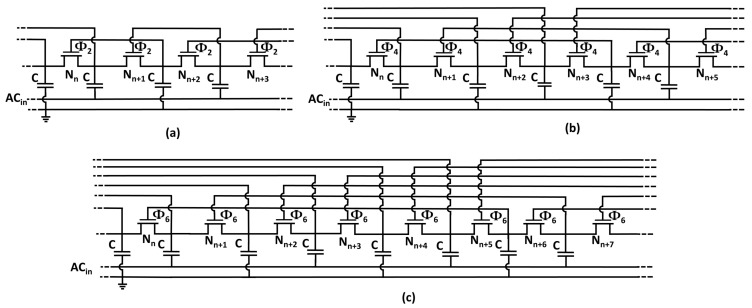
Generalized representation of a voltage multiplier with (**a**) a second order of compensation, Φ=2, (**b**) a fourth order of compensation, Φ=4, and (**c**) a sixth order of compensation, Φ=6.

**Figure 7:**
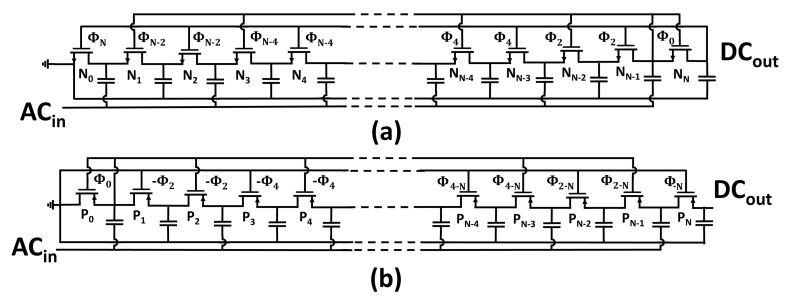
Voltage multiplier employing (**a**) NMOS and (**b**) PMOS transistors in the rectification chain, implemented maximizing the compensation order.

One category of threshold-cancellation techniques is based on a self-compensation approach first disclosed in a patent filed by Dickson in 1980 [27]. The threshold reduction is achieved by connecting the gate of NMOS transistors to ensuing pumping nodes in the multiplier chain. The $V_{TH}$-reduction technique can be also implemented through backward gate connections for a Positive-Channel Metal Oxide Semiconductor (PMOS) rectifier topology. The advantage of the aforementioned $V_{TH}$-compensation approach lies in the profitable exploitation of inherent properties of voltage waveforms in the inner nodes of the Dickson rectifier. The dc voltage component in the Dickson multiplier raises progressively through the rectifier chain from the ac input to the dc output. Therefore, the bias voltage can be found in successive nodes of the multiplier chain. An analytical estimation of the bias voltage generated by employing this threshold-compensation technique is given by:

(5) $$V_{bias}=\frac{\Phi }{2}\left[\frac{C}{C+C_{P}}V_{AC}-\frac{I_{OUT}}{f(C+C_{P})}-V_{TH}\right],$$

where Φ is the order of the compensation, which indicates the distance of the gate connection from ensuing or preceding pumping nodes, as shown in Figure 6. In Dickson-based voltage doublers (VDs) relying on a single power source, only even compensation orders are allowed due to the presence of alternating phases in the inner nodes of the rectification chain. Under the assumption of a multiplier chain implemented by maximizing the compensation order (shown in Figure 7), the bias voltage expression can be rewritten as:

(6) $$V_{bias}=\frac{\Phi }{2}\left[\frac{C}{C+C_{P}}V_{AC}-\frac{I_{OUT}}{f(C+C_{P})}-\left(V_{TH}-V_{bias}\right)\right].$$

Equation (6) entails a new and less-rigid input voltage condition given by:

(7) $$V_{AC}>\frac{C+C_{P}}{C}\left(V_{TH}-V_{bias}\right)+\frac{I_{OUT}}{fC},$$

to be satisfied in order to attain a positive bias and output voltage, thus reducing the input dead-zone of voltage multipliers.

**Figure 8:**
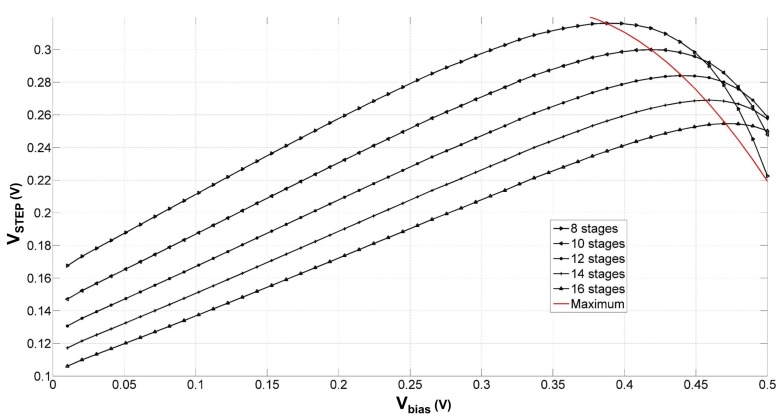
Simulated average step voltage versus the static bias voltage for multiple voltage multipliers differing in the number of stages. ($V_{AC}$ = 500 mV, *C* = 3 pF, *L* = 90 nm, *W* = 15 μm, *f* = 1 GHz, $R_{OUT}$ = 1 MΩ).

Based on the approach first disclosed in patent [27], all the self-compensated passive $V_{TH}$-compensation techniques generate a static bias voltage to reduce the threshold voltage of transistors in the rectification chain. The simulated average step voltage, expressed as:

(8) $$\begin{array}{cc} V_{STEP}=\frac{\sum_{n=1}^{N}\left(V_{n}-V_{n-1}\right)}{N} & \mathrm{with}V_{0}=0 \end{array}$$

versus the bias voltage for multiple compensated voltage multipliers differing in the total number of stages is depicted in Figure 8.

The simulations were conducted by connecting an ideal dc source at the input of each transistor gate, as depicted in Figure 5. The width of the transistors in the multiplication chain and the coupling capacitors were not scaled along the rectification chain. Although the output voltage increases with the number of multiplier stages, the maximum step voltage increment across each node tends to slowly saturate. Furthermore, the number of stages and the optimum bias voltages increase concomitantly.

The decreasing output voltage behavior is due to the exponential dependence of the reverse leakage current (or sub-threshold current) to the bias voltage applied at the gate of the transistors in the multiplier chain [28]. The bias voltage increases both the reverse current and the forward one; however, the amplitude increment of the latter is slower than the amplitude sweep of the sub-threshold current [18]. Furthermore, the time period at which the voltage multiplier endures the leakage current corresponds to more than half of a rectifier conduction cycle [29]. As formulated in Equation (5), the static bias voltage expression entails a linear dependence on the peak-to-peak amplitude voltage of the input signal $V_{AC}$, and hence an exponential dependence on the leakage current when the transistors are operated in sub-threshold. An analytical estimation of the forward current $I_{DS}$ as a function of the bias voltage can be expressed as follows [17]:

(9) $$I_{DS}=I_{Sat}\left[\exp\left(\frac{V_{AC}+V_{bias}}{V_{T}}\cos\left(ft\right)\right)\exp\left(\frac{V_{OUT}}{2NV_{T}}\cos\left(ft\right)\right)-1\right]+C\frac{\delta V_{DS}}{\delta t},$$

where $V_{T}$ is the thermal voltage and $I_{Sat}$ is the saturation current, which—assuming a constant dc voltage increment along the NMOS multiplier chain—can be derived as:

(10) $$I_{Sat}=\frac{\mu_{n}C_{OX}W_{n}{\left(V_{GS}-V_{TH}+V_{bias}\right)}^{2}\left(1-\lambda V_{DS}\right)}{2L_{n}}.$$

## 3. Prior Art Threshold-Compensation Techniques

**Figure 9:**
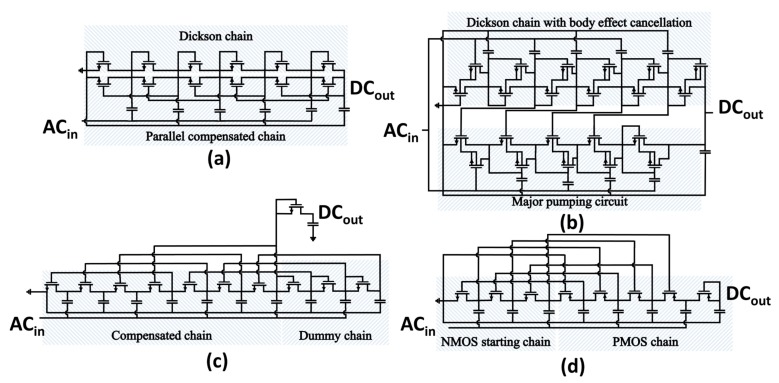
Prior-art threshold-compensation techniques for multi-stage voltage multipliers. (**a**) $V_{TH}$-reduction technique through charge transfer switches [30]. (**b**) Threshold-compensation through body effect reduction and parallel compensated chain [14]. (**c**) $V_{TH}$-cancellation technique through a constant order forward compensated NMOS chain [18]. (**d**) $V_{TH}$-compensation technique realized with NMOS and PMOS transistors forward and backward compensated, respectively [16].

Some of the prior-art passive (in this paper, the term passive is reserved for threshold-compensation approaches relying only on the input power source available during normal operating condition; therefore, the threshold-cancellation technique proposed in [25] is not considered as passive because charge was injected in the device to program floating-gate transistors) threshold-compensation techniques based on [27] are summarized in Figure 9. Voltage multipliers shown in Figure 9a,b are contextualized to rectify a single ac signal by grounding one of the two non-interleaving clock signals analogously to the Dickson multiplier topology shown in Figure 3.

The first approach, shown in Figure 9a, consists of paralleling node-by-node the Dickson multiplier to charge transfer switches (CTSs) comprising the higher-order compensation chain [30]. As the peak-to-peak ac voltage approaches the transistor threshold voltage, the higher-order compensation chain operates in the triode region, whereas the diode-connected transistor chain is still operated in the sub-threshold region or in cutoff mode. The choice of a double rectifying chain entails an increased circuit area occupation, and hence there is a possible argument for power dissipation.

An optimization of the CTS technique is shown in Figure 9b. The objective is to increase the efficiency of the multiplier by reducing the body effect through small MOSFET transistors connected to the substrate of the main pumping devices. Another improvement is given by the unbalanced width and number of transistors in the two chains. Analogously to the previous case, the dual-chain constitutes the main limitation of the proposed $V_{TH}$-reduction approach.

The threshold-compensation technique proposed by Papotto et al. in [18] is shown in Figure 9c. The rectifier was designed generalizing the same working principle exploited by the aforementioned techniques, proposing higher compensation orders depending on the intended threshold reduction. Unlike all of the previously-discussed approaches, the proposed threshold-rectification comprises only a single multiplication chain implemented in standard triple-well CMOS technology. The use of a single chain implies a reduced number of components, and consequently a reduction in the physical area occupation. The IC area overhead is mainly due to the capacitors and transistors in the dummy chain. The pumping nodes in the rectifier termination chain are solely employed to generate the bias voltage for the threshold-compensation technique. Therefore, higher voltage levels generated by the termination chain are not supplied to the load.

Hameed et al. in [16,31] proposed a threshold-compensation technique (Figure 9d) to overcome the limitations imposed by the dummy chain in the rectifier design. The technique uses both forward-compensated NMOS transistors and backward-compensated PMOS transistors in a single multiplication chain. The dual choice of NMOS and PMOS transistors allows full exploitation of each pumping node in the rectification chain. Thereby, the output voltage can be directly attained at the output of the last pumping node, which is implemented by an uncompensated diode-connected PMOS transistor to reduce the leakage losses. To maintain the incremental voltage constant across the multiplier chain, proper scaling of the pumping cells was adopted.

## 4. Lower-Order Compensation Chain

**Figure 10:**
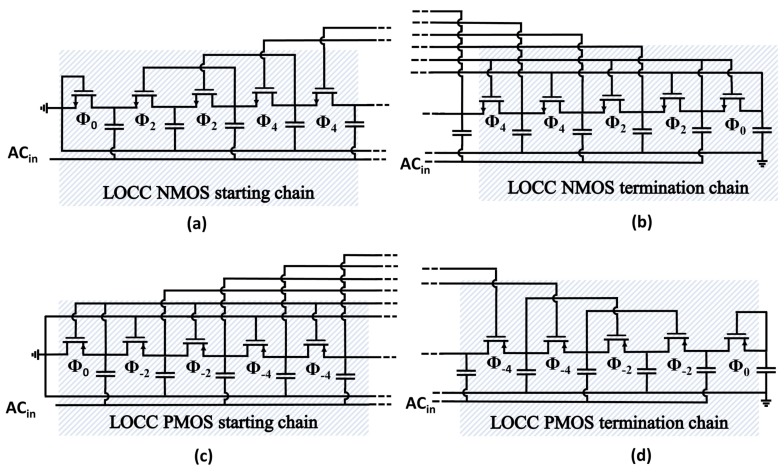
Circuit schematic of the proposed threshold-reduction technique implemented employing (**a**) an NMOS starting chain; (**b**) an NMOS termination chain; (**c**) a PMOS starting chain; and (**d**) a PMOS termination chain. LOCC: lower-order compensation chain.

The lower-order compensation chain (LOCC) was devised to allow better control of the trade-off among transistor conductivity and leakage losses through a gradual degradation of the produced bias levels and transistor conductivity. The technique consists of a linear and dual reduction in the compensation order in the starting or termination chain, as shown in Figure 10. Unlike all of the threshold-reduction techniques presented in Section 3, the LOCC employs several compensation orders in a single multiplication chain. The order of compensation in the starting or termination chain can be finely tuned according to the intended threshold reduction during the rectifier design.

Voltage multiplier topologies will be also characterized in a tabular form, where the transistors in the rectification chain are represented with the relative compensation order. An example of a tabular form representation pertaining to the circuit topology proposed in Figure 7 is shown in Table 1.

To estimate the step voltage expression for a given compensated rectifier topology, the concept of rectifier class Ψ is introduced as:

(11) $$\Psi =\sum_{n=1}^{N}\Phi_{n},$$

where *n* is the transistor index. The average compensation order $\bar{\Phi }$, expressed as:

(12) $$\bar{\Phi }=\frac{\Psi }{N}$$

will also be adopted in the following derivations.

Both parameters can assume positive and negative values to characterize forward or backward connections, respectively. Hence, a voltage multiplier topology comprising either NMOS or PMOS transistors in the rectifier chain can be uniquely identified by one of the two couplets (Ψ,*N*), ($\bar{\Phi }$,*N*).

The average compensation order can be normalized as:

(13) $$\Delta =1-\frac{2|\Phi |}{N}.$$

The Δ coefficient is a real and positive number which can assume values between 0, for a rectifier with a maximized compensation chain, and 1, for an uncompensated voltage multiplier.

Assuming a suitably circumvented body effect and a constant voltage increment across each multiplier stage during steady state operation, the output voltage can be expressed as:

(14) $$V_{OUT}=\frac{\alpha \beta N}{2\beta +N}V_{AC}-\frac{N\beta -2\beta }{2\beta +N}V_{TH},$$

where α and β are two dimensionless coefficients given by:

(15) $$\begin{array}{cc} \alpha & =\frac{C}{C+C_{p}},\\ \beta & =R_{OUT}f\left(C+C_{p}\right), \end{array}$$

where $R_{OUT}$ is the resistance seen by the last pumping node in the rectifier chain. Equation (14) is attained expressing the output current as the product of the output conductance and the output voltage, and by eliciting the latter from Equation (3).

Assuming a constant voltage increment along the multiplication chain, the step voltage expression can be consequently derived considering the difference between two successive nodes (e.g., $V_{OUT_{N}}-V_{OUT_{N-1}}$). The resulting expression is given by:

(16) $$V_{STEP}=\frac{2\alpha \beta^{2}V_{AC}-\left(2\beta^{2}-2\beta \right)V_{TH}}{\left(2\beta +N)(2\beta +N-1\right)}.$$

Equation (16), which is derived from Equation (3), does not inherit the nonlinear voltage–current characteristic of transistors operated in the sub-threshold region. Consequently, the step voltage expression in Equation (16) might incorrectly assume negative values, depending on the difference between the peak-to-peak ac voltage and the transistor threshold voltage.

The step voltage behavior for different rectifier classes, explicated as:

(17) $$V_{STEP}=\frac{2\alpha \beta^{2}\Phi_{2}^{1}V_{AC}-\left(2\beta^{2}-2\beta \right)\Phi_{2}^{2}V_{TH}}{\left(2\beta +N)(2\beta +N-1\right)},$$

avails from two sigmoidal correction parameters—$\Phi_{2}^{1}$ for the peak-to-peak ac input voltage and $\Phi_{2}^{2}$ for the threshold voltage—to correct the aforementioned unfitness, and to account for the compensation effect due to forward and backward gate connections. The two sigmoid functions, formalized as:

(18) $$\Phi_{2}^{1}=\frac{2\Delta -1}{8\pi }\arctan\left(\frac{V_{AC}-V_{TH}\Phi_{1}^{2}}{V_{TH}}\right)+\Phi_{1}^{1},$$

(19) $$\Phi_{2}^{2}=\frac{\Phi_{1}^{2}-\Delta }{8\pi }\arctan\left(\frac{V_{AC}-V_{TH}\Phi_{1}^{2}}{\Phi_{1}^{1}V_{TH}}\right),$$

are based on two positive and empirical parameters, $\Phi_{1}^{1}$ expressed as:

(20) $$\begin{array}{cc} \Phi_{1}^{1} & =\frac{1}{2}-\frac{1}{N}-\left(\sum_{n=\frac{N}{2}-3}^{\frac{N}{2}}\frac{1}{2n}\right)\;\;\mathrm{when}\;N\;\geq \;10,\\ \Phi_{1}^{1} & ={\left(\frac{1}{14-N}\right)}^{2}\;\;\mathrm{when}\;N\;<\;10. \end{array}$$

and $\Phi_{1}^{2}$, defined as:

(21) $$\Phi_{1}^{2}=1+\frac{1+\Delta }{2\Phi_{1}^{1}N},$$

which are two real and positive coefficients dependent on the total number of stages only. The effect of the total stage number on the step voltage will be further discussed in Section 5.

**Figure 11:**
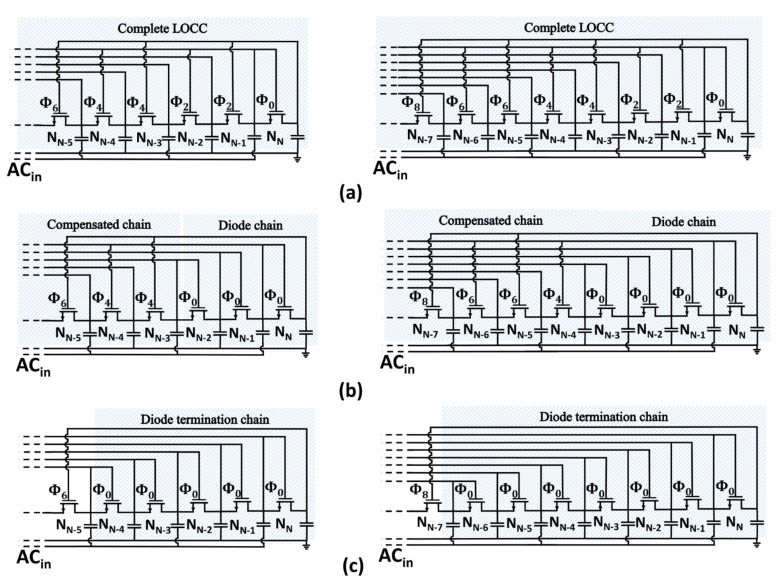
Circuit schematic of NMOS termination chains comprising: (**a**) a complete LOCC, (**b**) a diode chain and a compensated chain, (**c**) only diode-connected transistors.

Equations (18) and (19) describe how the normalized average compensation order impacts the step voltage. The advantage of the LOCC is that the average compensation order can be finely tuned by changing the number of diode-connected transistors, as depicted in Figure 11.

**Figure 12:**
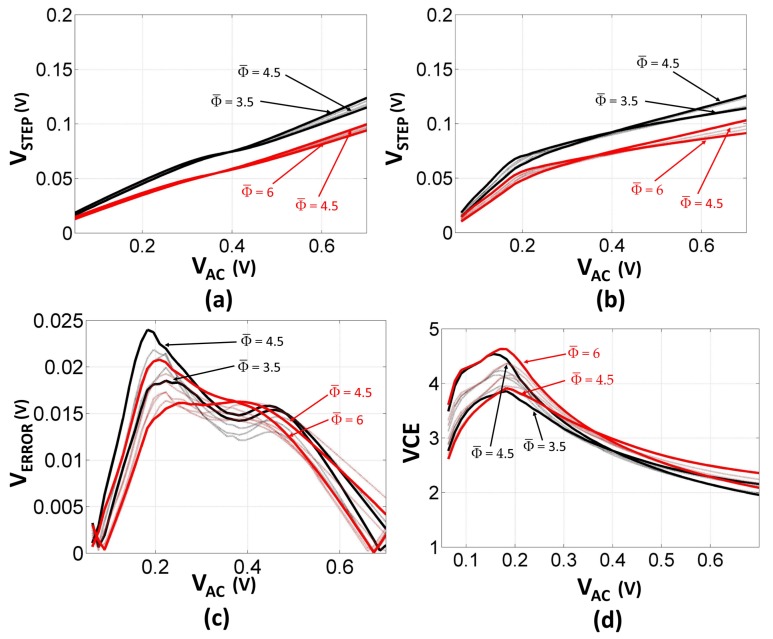
Voltage waveforms for (**a**) the modeled and (**b**) the simulated step voltage versus $V_{AC}$, with (**c**) error characterization for the proposed model, and (**d**) simulated voltage conversion efficiency (VCE) for the different compensation techniques. (*N* = 16 with $|\bar{\Phi }|$ = 6, 5.875, 5.75, 5.5, 5.25, 4.875, 4.5; *N* = 12 with $|\bar{\Phi }|$ = 4.5, 4.333, 4.166, 3.833, 3.5; *C* = 3 pF; L = 95 nm; W = 12 μm; *f* = 1 GHz).

The proposed LOCC technique and the relative model were validated and tested considering 12 different voltage multipliers, as detailed in Table 2. The simulation and modeling results for the proposed topologies are summarized in Figure 12. The simulated voltage multipliers were implemented assuming an n-well fabrication process.

The number of diode-connected transistors in the LOCC affects the step and output voltage behavior of the rectifier, as shown in Figure 12a. At lower peak-to-peak ac voltage levels, rectifiers with a higher average compensation order attain a higher output voltage and VCE. Conversely, at high input voltage levels, voltage multipliers implemented with a lower average compensation order exhibit a better output asymptotic behavior.

The VCE is shown in Figure 12d. From Figure 12b, it can be noticed that voltage multipliers implemented with a lower number of stages attain a higher step voltage, whereas when considering the overall output voltage, rectifiers with a higher stage number generally achieve a better performance. Note that t he output voltage behavior is strongly dependent on the compensation order. Therefore, for certain peak-to-peak values of $V_{AC}$, rectifiers with a higher number of stages and a higher average compensation order might be outperformed by topologies with a lower total stage number and a lower average compensation order.

## 5. Comparison of Threshold-Reduction Techniques

In the following discussion, threshold-compensation techniques will be compared considering the rectifier class and physical area occupation approximated by the total number of pumping stages in the multiplier chain. The parallel Dickson chain proposed by the two implementations depicted in Figure 9a,b does not affect the resulting rectifier class ($\bar{\Phi }=0$). Therefore, the two $V_{TH}$-reduction techniques proposed in [14,30] will not be considered in the following comparison.

The rectifier class expression for a voltage multiplier implemented with a maximized compensation chain (i.e., the circuit topology represented in Figure 7 and Table 1) is given by

(22) $$|\Psi_{MAX}|=\frac{N^{2}}{2}.$$

To derive the following class expressions, an equal number of pumping stages employed in the diode-connected chain, in the NMOS starting chain, in the PMOS termination chain, and in the LOCC is assumed.

Premising *N*≥ 4 and *N* mod 2 = 0, the maximum rectifier class formulation relative to the circuit topology proposed in [18] can be derived as:

(23) $$\begin{array}{cc} \left|\Psi_{Papotto}\right| & =\frac{N^{2}+2N}{4}\;\;\mathrm{when}\;N\;\mathrm{mod}\;4\;=\;0,\\ \left|\Psi_{Papotto}\right| & =\frac{N^{2}+2N-8}{4}\;\;\mathrm{when}\;(N-2)\;\mathrm{mod}\;4\;=\;0. \end{array}$$

The rectifier class expression pertaining to the threshold-compensation technique proposed in [16] can be expressed as:

(24) $$\begin{array}{cc} \left|\Psi_{Hameed}\right| & =\frac{N^{2}}{2}\;\;\mathrm{when}\;N\;\mathrm{mod}\;4\;=\;0,\\ \left|\Psi_{Hameed}\right| & =\frac{N^{2}-2N}{2}\;\;\mathrm{when}\;(N-2)\;\mathrm{mod}\;4\;=\;0, \end{array}$$

whereas the modulus of the LOCC class can be formulated as:

(25) $$\begin{array}{cc} \left|\Psi_{LOCC}\right| & =\frac{3N^{2}}{8}\;\;\mathrm{when}\;N\;\mathrm{mod}\;4\;=\;0,\\ \left|\Psi_{LOCC}\right| & =\frac{3N^{2}-4N-4}{8}\;\;\mathrm{when}\;(N-2)\;\mathrm{mod}\;4\;=\;0. \end{array}$$

The compensation order modulus was considered in the derivation of the Hameed rectifier class expression, because the Hameed topology comprises both backward and forward gate connections in the rectifier chain.

**Figure 13:**
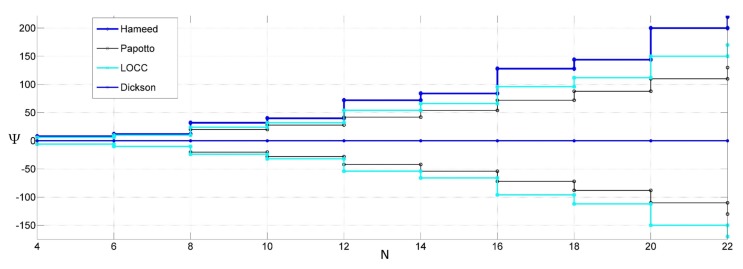
Rectifier class versus the number of stages for different threshold-compensation techniques.

The class expressions versus the number of stages are plotted in Figure 13. The rectifier class gives a preliminary indication of the expected threshold reduction.

It is important to note that the intended threshold reduction design is influenced by the number of multiplication stages, as depicted in Figure 8. Therefore, the rectifier design should account for the mutual influence of the rectifier class on the number of stages Ψ(N)⇌N(Ψ), inclining towards higher classes of rectification for implementations employing higher numbers of stages.

## 6. Experimental Verification

The conducted experiments were aimed at validating the theoretical conjectures presented in Section 4. The LOCC was verified by comparing the proposed technique with the $V_{TH}$-cancellation technique introduced in [18] and the uncompensated Dickson rectifier.

The five prototypes (6-stage, 8-stage, 10-stage, 12-stage, and 14-stage) were realized by employing 2N5247 *N*-channel junction field effect transistors (JFETs) [32] and 470 nF 104 M ceramic capacitors wire-bounded to FR4 printed circuit boards (PCBs). Each PCB included the three different multi-stage rectifier implementations. The two threshold-compensation techniques for the four prototypes were implemented congruously to Equations (23) and (22).

The measurements were attained by considering a single-tone sinusoidal continuous-wave 50-Ω source with a nominal frequency of 100 kHz and a 3.9 MΩ resistive load. The output voltage of the three implementations was measured at the last multiplier stage.

**Figure 14:**
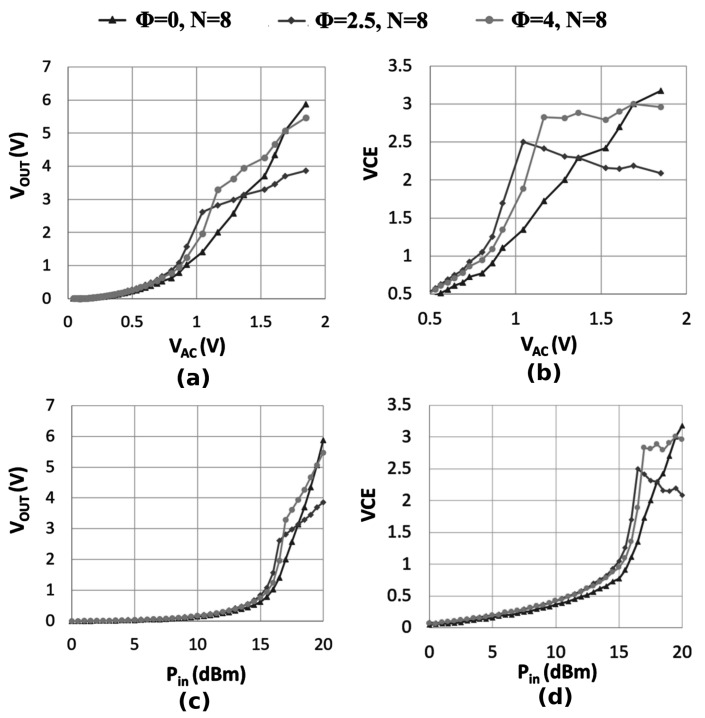
Measured output voltage and VCE versus the input power ($P_{in}$) and the peak-to-peak input voltage ($V_{AC}$) for different voltage multiplier topologies. ($R_{OUT}$ = 3.9 MΩ, *C* = 470 nF, *f* = 100 kHz).

Figure 14 plots the measured output voltage and VCE versus the input power and voltage, respectively, for three 8-stage voltage multipliers. The attained measurements agree with the theoretical conjectures and simulations discussed in Section 4, asserting the output characteristic of compensated and uncompensated voltage multipliers. Considering the VCE and the output voltage characteristic when the input power is below 16.5 dBm, the LOCC outperformed both rectifiers with a lower average compensation order.

**Figure 15:**
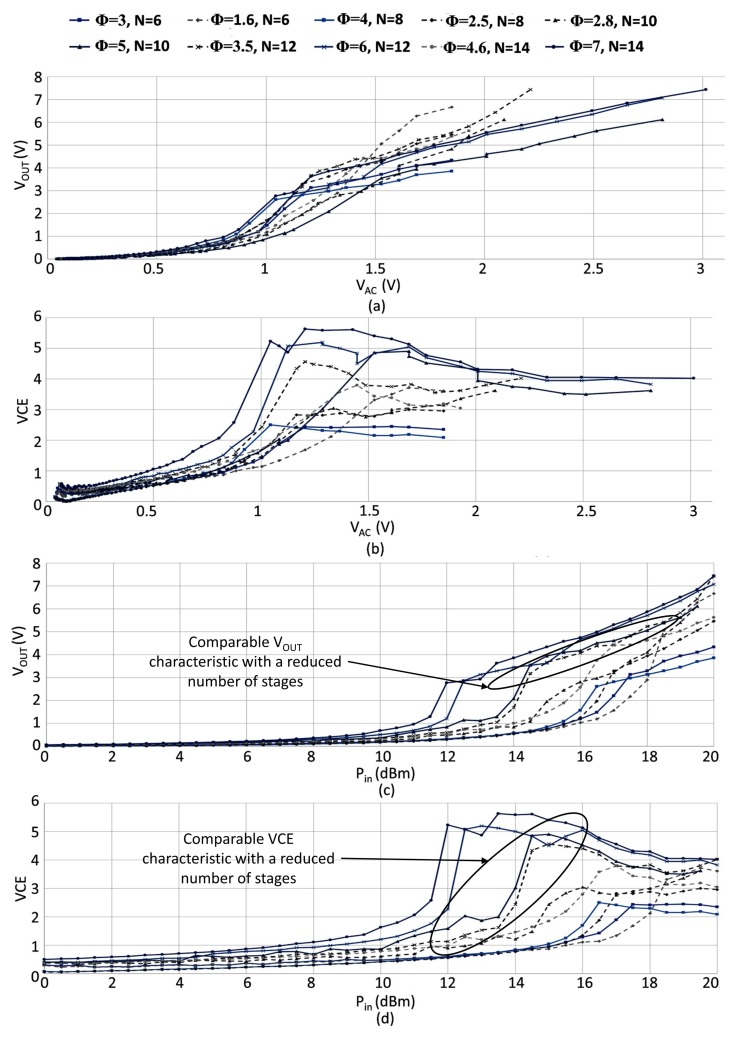
Measured waveforms partaining to (**a**) output voltage versus the peak-to-peak input voltage, (**b**) VCE versus the peak-to-peak input voltage, (**c**) output voltage versus the input power ($P_{in}$) and (**d**) VCE versus the input power. ($R_{OUT}$ = 3.9 MΩ, *C* = 470 nF, *f* = 100 kHz).

The divergence in the attained results for the two threshold compensation techniques increased with the order of compensation and the number of stages, as shown in Figure 15.

This property resulted in a reduced physical area occupation for a given target performance, where a 10-stage multiplier realized by employing the LOCC attained similar performance to a 12-stage rectifier realized with a constant compensation order if the input power was below 17 dBm.

## 7. Conclusions

This paper introduced a threshold-compensation technique for multi-stage voltage multipliers. The proposed $V_{TH}$-reduction technique enables a better control of the trade-off among transistor conductivity and leakage losses through a topological design methodology, which is aimed at optimizing the VCE for a given input signal and target output performance. The effectualness of the threshold-compensation approach was verified and validated through simulations, asserting the theoretical derivations for the proposed rectifier model. An experimental validation and comparison of threshold-compensation techniques was performed, and the attained measurements clearly support the effectiveness of the LOCC, which can effectively reduce the voltage multiplier physical area occupation for a given target performance and ac input signal compared to previous art $V_{TH}$-reduction techniques. Future work will comprise the design, realization, and testing of an IC for the proposed $V_{TH}$-reduction technique.

## Figures and Tables

**Table 1:** Tabular representation of two *N*-stage voltage multipliers employing maximized compensation chains.

Chain Topoogy	Rectifier Tabular Form											
NMOS chain	*N*	N−2	N−2	N−4	N−4	...	4	4	2	2	0	
PMOS chain	0	−2	−2	−4	−4	...	4−N	4−N	2−N	2−N	−N	

**Table 2:** Tabular form of the simulated voltage multipliers.

Voltage Multiplier Topology										
N	Ψ	$\bar{\Phi }$	**Rectifier Tabular Form**							
16	96	6	8×9	6	6	4	4	2	2	0
16	94	5.875	8×9	6	6	4	4	2	0×2	
16	92	5.75	8×9	6	6	4	4	0×3		
16	88	5.5	8×9	6	6	4	0×4			
16	84	5.25	8×9	6	6	0×5				
16	78	4.875	8×9	6	0×6					
16	72	4.5	8×9	0×7						
12	54	4.5	6×7			4	4	2	2	0
12	52	4.3333	6×7			4	4	2	0×2	
12	50	4.1667	6×7			4	4	0×3		
12	46	3.8333	6×7			4	0×4			
12	42	3.5	6×7			0×5				
